# What Is the Rate of Antimicrobial Resistance of a Prosthetic Joint Infection in a Major Orthopaedic Centre?

**DOI:** 10.3390/antibiotics13040306

**Published:** 2024-03-28

**Authors:** Belgin Coskun, Müge Ayhan, Merve Bozer, Halil Ibrahim Ozaslan, Metin Dogan, Mustafa Citak, Mustafa Akkaya

**Affiliations:** 1 Infectious Diseases and Clinical Microbiology, Ankara Bilkent City Hospital, Ankara 06800, Turkey; belgintekin@yahoo.com (B.C.); dr.mugeayhan@hotmail.com (M.A.); 2 Department of Orthopaedics & Traumatology, Ankara Yıldırım Beyazıt University, Ankara 06800, Turkey; dr.bozermerve@gmail.com (M.B.); dr.h.ibrahim.ozaslan@gmail.com (H.I.O.); drmetindogan@gmail.com (M.D.); 3 Department of Orthopaedic Surgery, Helios ENDO-Klinik Hamburg, 22767 Hamburg, Germany; mcitak@gmx.de; 4 Department of Orthopaedics & Traumatology, Ankara Guven Hospital, Ankara 06540, Turkey

**Keywords:** antibiotics, arthroplasty, infection, periprosthetic joint infection, revision arthroplasty

## Abstract

Periprosthetic joint infections (PJIs) are important factors in decreasing the success of hip and knee arthroplasties. It is a necessity to explore the epidemiological data and develop applications for rational antibiotic use, to address future infection control concerns. We aimed to investigate the microorganisms that were responsible and the related antibiograms in 121 patients with PJI, who were managed by two-stage revision surgery. Patients’ data records, demographics, comorbidities, sites of arthroplasty, synovial fluid and deep tissue culture results and antibiotic treatment were summarized on a standardized case report form. There were 43 (35.5%) culture-negative PJI cases and 12 (9.9%) polymicrobial growths. The causative pathogens included Gram-positive (50.4%) and Gram-negative microorganisms (23.1%) and fungi (0.8%). Methicillin resistance was 64.3% for S. aureus and 89.5% for coagulase-negative staphylococcus (CoNS). The extended spectrum beta lactamase (ESBL) rate for Enterobacteriaceae was 68.4%. This study shows that antibiotic resistance is encountered in more than half of the cases, which is valid for all microorganisms most common in PJI. The success of treatment decreases significantly in cases where antibiotic-resistant microorganisms are isolated or in cases where the culture is negative.

## 1. Introduction

Knee and hip arthroplasty surgeries are largely successful elective surgical procedures with a 10-year follow-up survival rate of over 95% [1]. However, periprosthetic joint infections (PJIs) are one of the most important factors in lowering this success [2], and so much so that the presence of infection ranks first among the reasons for revision after knee arthroplasty with a rate of 25.2%, and accounts for 14.8% of revisions after hip arthroplasty [3,4]. There are studies suggesting that PJIs occur at a rate of 1% per year for hip arthroplasties and between 1% and 2% per year after knee arthroplasties [5,6].

Antibiotic administration is the standard practice for both prophylaxis and treatment against PJI after joint arthroplasties [7]. However, the Centers for Disease Control (CDC) reports that the majority of all antibiotics prescribed against infections after joint arthroplasties in acute care hospitals are unnecessary or inappropriate [8]. This practice may lead to an increase in antibiotic resistance and may cause additional morbidities due to harmful side effects without providing any benefit to the patient. Therefore, optimizing the selection, dose and duration of antibiotics is necessary for effective infection treatment and is important for preventing possible resistance development and undesirable side effects.

In a previous study, the most common organisms isolated from the infected joints were reported to be methicillin-resistant and methicillin-sensitive *Staphylococcus aureus* and methicillin-resistant and methicillin-sensitive *Staphylococcus epidermidis* [9]. A study conducted in Europe reported that 12% of *Streptococcus pneumoniae* strains had a decreased susceptibility to penicillin, more than 15% of *S. aureus* strains were resistant to methicillin, and approximately 9% of *Enterococcus* species had become resistant to vancomycin [10]. The rate at which microorganisms develop resistance and the lack of new antibiotic discoveries to keep up with this pace are worrying in terms of future infection control. It is an absolute necessity to explore the epidemiological data and develop applications for rational antibiotic use.

For this reason, in our study, we aimed to investigate our epidemiological data by examining the microorganisms responsible and the related antibiograms during follow-up of patients post joint replacement in our tertiary hospital in Turkey.

## 2. Results

One hundred and twenty-one patients were included in this study. Their demographic features are shown in Table 1. The median age was 68 years (IQR 12.0) and 37 (30%) were male. Of the patients, 74 (64.2%) had the knee as the PJI site, which was the most commonly infected site. Diabetes was the most common comorbidity (52.9%). Diabetes was followed by hypertension, rheumatoid arthritis, chronic renal failure and coronary artery disease, respectively. Forty-three patients (35.5%) had a previous history of PJI. Seventy-six patients (62.8%) had a history of antibiotic use in the last 3 months. The majority of the patients had late PJI (66.9%). Twenty-four of the patients had delayed PJI and 9.1% had early PJI.

There were 43 (35.5%) culture-negative PJIs in our study group. Twenty-three (53.4%) of the culture-negative PJI patients had a history of antibiotic use. There were 90 causative pathogens in 78 patients. There were 12 (9.9%) polymicrobial growths. The causative pathogens included Gram-positive (50.4%) and Gram-negative microorganisms (23.1%) and fungi (0.8%) (Table 2). The most common causative microorganisms were *Staphylococcus aureus* (23.1%), coagulase-negative *staphylococci* (CoNS) (15.7%) and *Escherichia coli* (7.4%). Methicillin resistance was 64.3% for *S. aureus* and 89.5% for *CoNS* (Table 2). The ESBL rate for *Enterobacteriaceae* was 68.4% (Table 3).

The mean C-reactive protein (CRP) of PJI patients before prosthesis removal was 83.8 mg/dL (±80.486). The mean CRP before revision surgery was 6.4 mg/L (±3.549).

Empiric antibiotic therapy was started in all patients until the microorganism was isolated in an intraoperative culture. Monotherapy was given in 21 (17.4%) patients and combination therapy was given in 100 (82.6%) patients. Teicoplanin was the most frequently preferred antibiotic in empirical monotherapy. In combination therapy, the combination of teicoplanin, piperacillin and tazobactam was the most frequently preferred combination (Table 4). In 16 (13.2%) patients who received empirical antibiotic treatment, the initial antibiotics were revised because it was not effective against the isolated microorganism. In 62 (51.2%) patients, empirical treatment was appropriate for the isolated microorganism. However, due to the broad spectrum of empirical treatment, antibiotherapy was deescalated. The mean duration of antibiotic use after prosthesis removal was 34.86 ± 12.762 days.

## 3. Discussion

Our study is one of several large case series of PJI in the medical literature [11,12]. This study provides information about the local epidemiology of causative microorganisms in PJI in Turkey. Use of antibiotics before a surgical procedure may result in a false negative culture. So, for patients undergoing joint replacement surgery, it is recommended that the antibiotics be discontinued at least 2 weeks prior to the surgical procedure to detect the causative organism [13]. The patients in our study had stopped antibiotic treatment at least 2 weeks before. But, despite this, there were 43 (35.5%) culture-negative PJIs in our study group. In similar previous studies, the rate of culture-negative PJI ranged from 7 to 41% [11,12,14]. Culture negativity was associated most frequently with a history of previous antibiotic use [12,15]. Negative cultures may have resulted from previous antibiotic us, as was the case in 23 (53.4%) patients from our study who had used antibiotics. Other causes of culture negativity are the use of antibiotic-impregnated cement, biofilm development on the prosthesis surface, inappropriate culture medium and prolonged time to transfer the specimen to the laboratory [13]. In our study, patients received a two-stage revision and all the cultures we evaluated were specimens taken at the prosthesis removal stage. Therefore, patients did not have antibiotic-impregnated cement. Our hospital is a large campus, so it was thought that the lack of growth of anaerobic microorganisms might have been related to inappropriate culture media and prolonged transfer times.

Antibiotic resistance of microorganisms and treatment successes are shown in Table 3. In our study, the most common causative microorganism of PJI was *S. aureus*, followed by *CoNS*. In similar previous studies, *S. aureus* was the most common causative agent in PJI [11,12,16]. Methicillin resistance is a serious problem for *CoNS* and *S. aureus*. In our study, methicillin resistance was 64.3% for *S. aureus* and 89.5% for *CoNS*. It was observed that the success of treatment decreased significantly in cases where antibiotic-resistant microorganisms were isolated or in cases where the culture was negative (Table 3).

According to Okay et al., the rate of methicillin-resistant *staphylococci* was 24.3% between 2011 and 2017 [17]. A 2012 Australian study reported a 45% rate of methicillin-resistant *staphylococci* [18]. This rapid increase in antibiotic resistance over the years has become a significant problem in PJIs’ treatment [7]. Joint infections are a public health problem creating many challenges such as limited treatment options due to antibiotic resistance, prolong hospitalization as well as increased healthcare costs [19,20,21].

In our study, the rate of Gram-negative microorganisms in culture-positive PJIs was 23.1%. In previous studies, the rate of PJI caused by Gram-negative microorganisms was reported to be between 6% and 23% [22,23,24]. Especially in the treatment of culture-negative PJI, Gram-negative microorganisms should be kept in mind as well as Gram-positive ones during empirical treatments. The ESBL resistance rate for *Enterobacteriaceae* was 68.4% in our study groups. The treatment options in such cases are limited, especially for resistant Gram-negative infections, which may lead to treatment failure and long hospital stays [25].

Although the polymicrobial growth rate in PJI patients was 9.9%, the empirical combination antibiotic use rate was 82.6%. Empirical treatment should target *staphylococci* (including methicillin resistant) and Gram-negative *bacilli* [26]. Consistent with the literature, we mostly preferred teicoplanin and piperacillin–tazobactam in combination. In our study, the antibiotic regimen in 64.5% of cases was based on laboratory results. A lack of efficacy was the reason in 13.2% of cases, whereas, in 51.2% of cases, empiric antibiotic therapy appeared to be broad spectrum and, therefore, was deescalated. Our combination antibiotic use rate was high. Treatment of infected joints should not be delayed; therefore, we preferred broad-spectrum therapy and later deescalated as the isolated microorganisms were identified. In the two-stage prosthesis infection treatment, antibiotic use is recommended for 4–6 weeks after the prosthesis is removed [26]. In our study, the mean duration of antibiotic use was 34.86 ± 12.762 days. Our duration of antibiotic use is consistent with the guideline recommendation.

The limitations of our study include the retrospective design, which may have caused leaked data. By planning the study prospectively, risk factors for resistant microorganisms can be determined with a larger sample group. Despite the limitations, our study was conducted in the major orthopaedic centre of our country. Our orthopaedic centre is a 578-bed hospital within a large health complex with 4190 beds. Our hospital is a tertiary hospital and a reference centre that accepts patients from all over our country. So, this study contributes considerably to the country’s epidemiological data. Furthermore, we believe that these data will contribute to the empirical antibiotic selection for PJI.

## 4. Materials and Methods

Our retrospective study was conducted between January 2019 and December 2022. All cases of PJIs managed by two-stage revision surgery were evaluated in our tertiary reference hospital. Ethical approval was granted by our hospital’s Research Ethics Committee (approval date: 26 July 2023, approval number: E1-23-3831).

Patients were managed by infectious disease physicians and orthopaedic surgeons in a multidisciplinary team. Infected prostheses were removed and culture samples were taken from at least 3 different tissues during the operation. If the patient was receiving antibiotic treatment, it was discontinued at least 2 weeks before surgery to avoid a false negative culture. Patients who were <18 years old and had missing data were excluded from analysis. International Consensus Meeting 2018 criteria were used for the diagnosis of PJI. Persistent elevation of the erythrocyte sedimentation rate (ESR) (>30 mm/h), C-reactive protein (CRP) (>1 mg/dL) and D-dimer (>860 ng/mL) were considered as 1, 2 and 2 points, respectively. A synovial fluid white blood cell count >3000 cells/µL was given 3 points, an alpha defensin level higher than the cut-off value was given 3 points, a leukocyte esterase test (++) was given 3 points, a polymorphonuclear cell rate >80% was given 2 points and a synovial CRP value >6, 9 mg/L was considered as 1 point. Patients with >6 points were considered infected. Apart from this, the presence of 2 positive cultures or the development of a sinus tract at the wound site was directly considered as infection [27]. Classification according to the onset time was defined according to the study Coventry et al. [28]. Infections occurring after arthroplasty applications were classified into 3 groups according to their time of occurrence. Accordingly, Type 1 was defined as early postoperative infection (acute infection that occurs in the first 30 days after surgery), Type 2 was defined as late chronic infection (long-term infection that occurs >30 days after surgery) and Type 3 was defined as late hematogenous infection (two years after surgery).

All patients received intravenous empiric antimicrobial therapy in the postoperative period. If a microorganism was isolated in intraoperative cultures, the treatment was revised according to the antibiotic sensitivity pattern of the microorganism. If the microorganism could not be isolated from the culture, treatment was continued empirically.

Treatment success was defined as being antibiotic-free and symptom-free for at least one year after completing infection treatment. Treatment failure was defined as one of the following: the recurrence of the same joint infection with the same or a different microorganism within the first year; presence of acute inflammation in the periprosthetic tissue on histopathological examination or in any surgery performed within 1 year after infection treatment; the development of a sinus tract to the joint; or death from joint infection. Death of a patient due to sepsis in whom no other focus of infection other than joint infection was detected was defined as PJI-related death. Antibiotic history was defined as antibiotic use within the last 3 months. History of PJI was defined as previous infection in the same joint.

Data were collected from the medical records. Patients’ data records such as demographics, comorbidities, site of arthroplasty, synovial fluid and deep tissue culture results and administered antibiotic treatment were reviewed and summarized on a standardized case report form.

The identification of isolates in cultures was performed with a VITEK-2 (BioMeriux, Marcy-l’Étoile, France) automated identification device. Antibiotic susceptibility tests were performed automatically on the VITEK-2 (BioMeriux, France) identification device. Methicillin resistance was evaluated with cefoxitin. Resistance rates and MIC (minimal inhibitory concentration) values were determined according to EUCAST (The European Committee on Antimicrobial Susceptibility Testing) standards.

Descriptive statistical analysis was performed using IBM SPSS Statistics 25 software (SPSS Inc., Chicago, IL, USA, 2011). The assumptions of normality and homogeneity of variance were investigated by Shapiro–Wilk and Levene tests, respectively. Descriptive statistics were expressed as the mean ± standard deviation, median (25th percentile–75th percentile) or median (minimum-maximum) for continuous numerical variables, while categorical variables were expressed as the number of cases and %. The significance of the differences between the groups in terms of averages was analysed by Student’s t test, while the significance of the differences in terms of continuous numerical variables for which parametric test statistical assumptions were not met was evaluated by the Mann–Whitney U test.

Fisher’s exact test was used for the analysis of categorical data. On the other hand, the effect of the mean width of the thickest thigh on the development of PJI was investigated by univariate logistic regression analysis by calculating the odds ratios and 95% confidence intervals. *p* ≤ 0.05 results were considered statistically significant.

## 5. Conclusions

PJI treatment is difficult and requires teamwork. Adding to the challenge is antibiotic resistance, which causes difficulties in the treatment of joint infection, as in other infections. To combat the threat it poses, gaining knowledge of country and world epidemiological data is highly important for ensuring rational antibiotic stewardship. Gram-positive microorganisms are the most common pathogens for PJI, so they may be the focus; however, it is necessary to acknowledge that Gram-negative microorganisms are still the causative pathogen in one out of every five patients.

## Figures and Tables

**Table 1:** Baseline demographic and clinical characteristics.

**Age, median (IQR)**	68.000 (12.000)
**Sex, (male), n (%)**	37 (30.6)
**Comorbidities, n (%)**	
Diabetes	64 (52.9)
Hypertension	24 (19.8)
Rheumatoid arthritis	7 (5.8)
Chronic renal failure	4 (3.3)
Coronary artery disease	1 (0.8)
**Prosthetic joint site, n (%)**	
Knee	74 (61.2)
Hip	43 (35.5)
Shoulder	3 (2.5)
**PJI history, n (%)**	43 (35.5)
**Previous antibiotic use, n (%)**	76 (62.8)
**Polymicrobial growth, n (%)**	12 (9.9)
**Culture negative PJI, n (%)**	43 (35.5)
**PJI time, n (%)**	
Type 1 (≤1 mo)	11 (9.1)
Type 2 (>1 mo to ≤12 mo)	29 (24)
Type 3 (>12 mo)	81 (66.9)

Abbreviations: PJI; prosthetic joint infection.

**Table 2:** Distribution of isolated agents in patients with prosthetic joint infection.

**Microorganisms**	n (%)
**Gram-positive microorganisms**	61 (50.4)
*S. aureus*	28 (23.1)
-MRSA	18 (14.9)
Coagulase-negative staphylococci	19 (15.7)
-MRCoNS	17 (14.1)
*Streptococcus* spp.	8 (6.6)
*Enterococcus* spp.	3 (2.5)
*Bacillus* spp.	2 (1.7)
*Corynebacterium striatum*	1 (0.8)
**Gram-negative microorganisms**	28 (23.1)
**Enterobacteriaceae**	19 (15.7)
*E. coli*	9 (7.4)
*Klebsiella* spp.	6 (5.0)
*Serratia marcescens*	3 (2.5)
*Proteus mirabilis*	1 (0.8)
**Non-fermentative Gram-negative bacilli**	
*Pseudomonas aeruginosa*	4 (3.3)
*Burkholderia cepacia*	3 (2.5)
*Achromobacter* spp.	1 (0.8)
***Other***	
*Moraxella* spp.	1 (0.8)
***Fungi***	
*Scedosporium* spp.	1 (0.8)

Abbreviations: MRSA; methicillin-resistant Staphylococcus aureus, MRCoNS; methicillin-resistant coagulase-negative staphylococci.

**Table 3:** Distribution of methicillin and ESBL resistance, Gram-positive and -negative microorganisms, and polymicrobial and culture-negative cases, and their relationship with treatment success.

	**Cured Infection (n = 89)**	**Uncured Infection (n = 32)**	***p*-Value**
Methicillin resistance	22 (24.7)	12 (37.5)	0.096
ESBL resistance	7 (7.9)	6 (18.8)	0.687
Resistance to any antibiotic	30 (33.7)	20 (62.5)	0.005
Gram-negative microorganism	11 (12.4)	17 (53.1)	0.002
Gram-positive microorganism	44 (49.4)	17 (53.1)	0.020
Polymicrobial	5 (5.61)	7 (21.9)	0.008
Culture-negative	5 (15.6)	38 (57.3)	0.006

**Table 4:** Intravenous antimicrobial therapy used for prosthetic joint infections (n = 121).

	**Empirical Antibiotherapy, n (%)**		**Definitive Antibiotherapy, n (%)**	
**Monotherapy**	Teicoplanin	12 (1)	Teicoplanin	30 (24.8)
	Piperacillin–tazobactam	3 (2.5)	Meropenem	9 (7.4)
	Sulbactam ampicillin	3 (2.5)	Sulbactam ampicillin	5 (4.1)
	Meropenem	2 (1.7)	Ertapenem	3 (2.5)
	Daptomycin	1 (0.8)	Ceftriaxone	2 (1.7)
			Ceftazidime	1 (0.8)
			Piperacillin tazobactam	1 (0.8)
			Amoxicillin clavulanate	1 (0.8)
			Moxifloxacin	1 (0.8)
			Cefazolin	1 (0.8)
			Tigecycline	1 (0.8)
			Voriconazole	1 (0.8)
**Combination**	Teicoplanin + Piperacillin–tazobactam	53 (43.8)	Teicoplanin + Ciprofloxacin	57 (47.1)
	Teicoplanin + Ciprofloxacin	34 (28.1)	Teicoplanin + Meropenem	4 (3.3)
	Teicoplanin + Meropenem	8 (6.6)	Teicoplanin + Ceftriaxone	2 (1.7)
	Teicoplanin + Ciprofloxacin	5 (4.1)	Teicoplanin + Piperacillin–tazobactam	1 (0.8)
			Meropenem + Amikacin	1 (0.8)

## Data Availability

The data presented in the study are available on request from the corresponding author.
